# Supplementary figures and images for: Impact of the 3% Oxygen Desaturation Index via Overnight Pulse Oximetry on Cardiovascular Events and Death in Patients Undergoing Hemodialysis: A Retrospective Cohort Study
Source: J Clin Med. 2023 Jan 20;12(3):858. doi: 10.3390/jcm12030858 (PMC9917943; doi:10.3390/jcm12030858)

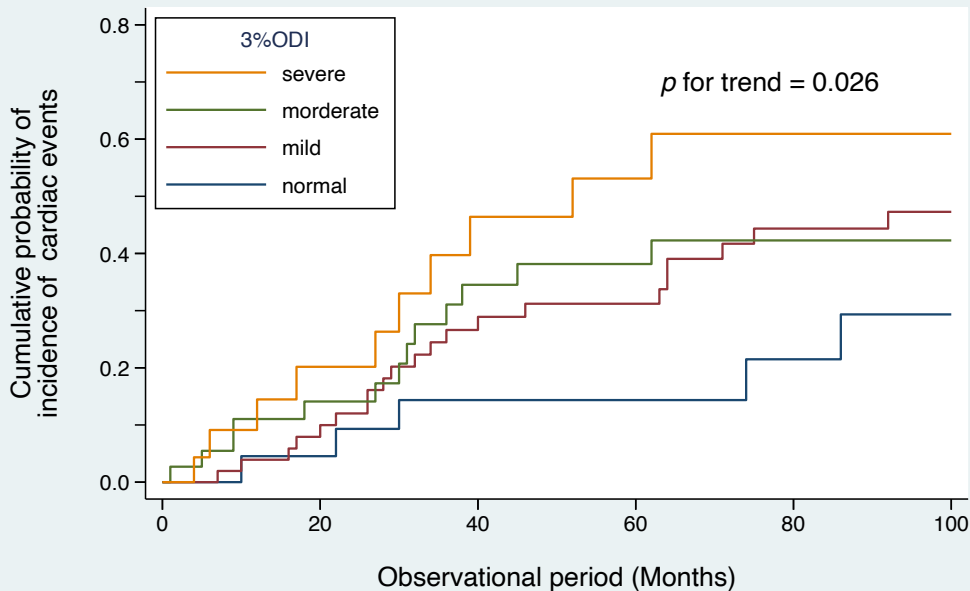

Number at risk

|           |    |    |    |    |    |    |
|-----------|----|----|----|----|----|----|
| normal    | 22 | 21 | 17 | 14 | 10 | 9  |
| mild      | 52 | 45 | 32 | 27 | 21 | 16 |
| morderate | 37 | 28 | 19 | 15 | 13 | 10 |
| severe    | 23 | 14 | 8  | 6  | 5  | 4  |

Supplement: Supplementary file 1 [file jcm-12-00858-s001.zip › Figure S1.pdf]

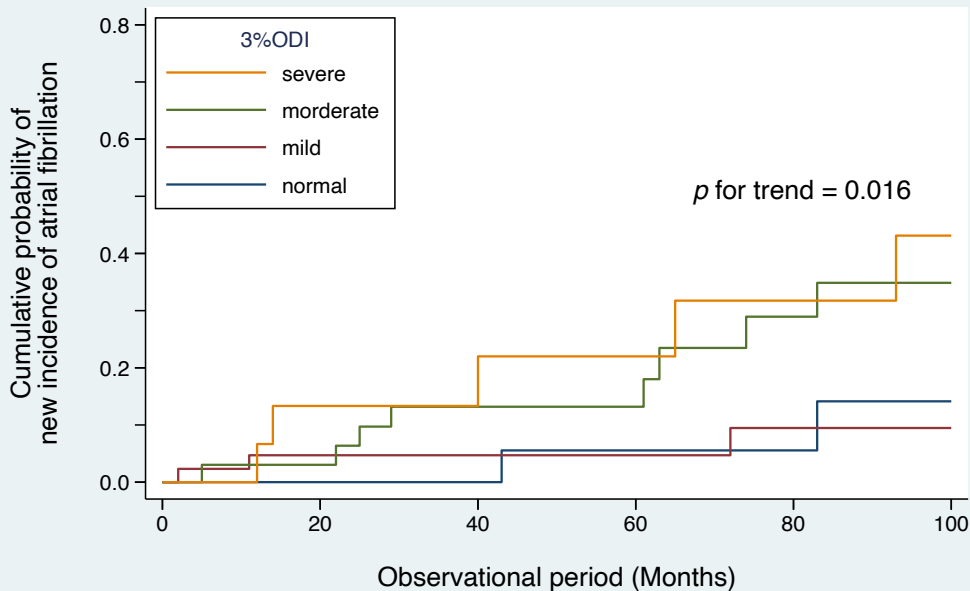

Number at risk

|           |    |    |    |    |    |    |
|-----------|----|----|----|----|----|----|
| normal    | 20 | 20 | 18 | 14 | 11 | 9  |
| mild      | 43 | 38 | 31 | 25 | 19 | 15 |
| morderate | 33 | 29 | 21 | 18 | 12 | 9  |
| severe    | 17 | 12 | 10 | 8  | 6  | 5  |

Supplement: Supplementary file 1 [file jcm-12-00858-s001.zip › Figure S2.pdf]
